# Success rate of IR midazolam sedation in combination with C-CLAD in pediatric dental patients—a prospective observational study

**DOI:** 10.7717/peerj.254

**Published:** 2014-03-06

**Authors:** Malka Ashkenazi, Anat Baniel

**Affiliations:** 1 Pediatric Dentist, Private practice, Petach-Tikva, Israel; 2 Beit Issie Shapiro’s Sensory Dental Clinic, Raanana, Israel

**Keywords:** Apprehensive, Local anesthesia, Dental, Children, Uncooperative

## Abstract

**Objective.** To evaluate the success rate of intra-rectal (IR) midazolam in combination with nitrous oxide/oxygen (N_2_O) sedation in young uncooperative dental patients when the local anesthesia is delivered by a computerized controlled local anesthetic delivery (C-CLAD).

**Study Design.** This observational study consisted of 219 uncooperative children (age: 4.3 ± 1.69 y) who received IR midazolam (0.4 mg/kg) and N_2_O to complete their dental treatment. Measured variables included: child’s pain disruptive behavior during delivery of anesthesia by C-CLAD (CHEOP Scale), child behavior during treatment (Houpt scale), dental procedure performed, and side effects that appeared during treatment.

**Results.** There was a high level of cooperation (mean score: 6.69 ± 2.1) during administration of local anesthesia. Good-to-excellent behavior was shown by 87% of the children during treatment. Planned treatment was completed by 184 (92%) patients. No statistically significant changes were noticed in the oxygen saturation levels before and after treatment. Children with side effects included 3 (1.3%) with nistagmus, 5 (2.3%) with diplopia, and 18 (8.2%) with hiccups. Three consecutive sedations decreased the overall behavior score by 5.7% compared to the first appointment (*p* < .05).

**Conclusions.** IR midazolam-N_2_O sedation in combination with C-CLAD is very effective for delivery of dental treatment to young uncooperative children.

## Introduction

Midazolam, a popular sedative agent used in pediatric dental offices, is effective and reliable in reducing anxiety in young children during dental treatment (Lindh-Stromberg, 2001; Jensen, Schroder & Mansson, 1999; Erlandsson et al., 2001; Fuks et al., 1994; Shapira et al., 1996; Lourenço-Matharu & Roberts, 2010; Coventry, Martin & Burke, 1991). Yet, its effectiveness is limited to 30%–85%, depending on the age of the child, the dose and the route of administration of the agent as well as on the skill of the dentist. Midazolam’s effectiveness as a sedative agent for performing dental treatment has been evaluated in children, aged 16 months to 10.5 years (Lindh-Stromberg, 2001; Jensen, Schroder & Mansson, 1999; Erlandsson et al., 2001; Fuks et al., 1994; Shapira et al., 1996; Lourenço-Matharu & Roberts, 2010; Coventry, Martin & Burke, 1991). Complete success was found in 39%–85% of the children, partial (by physical restraint) in 30%–61%, and no success (no treatment) in 0.4%–7%. Moreover, consecutive sedations were associated with decreased cooperation in 30% of the children (Erlandsson et al., 2001; Fuks et al., 1994; Shapira et al., 1996).

Midazolam for conscious sedation in the dental office can be administered orally, intra-nasally or rectally (IR). Uncooperative young children usually refuse to swallow the drug and may expectorate it, while intra-nasal administration is usually associated with painful burning sensation in the nasal mucosa that results in crying. For these children, administration of the midazolam rectally may be considered. Although some parents refuse this route, many others use it routinely for administrating analgesics to their young uncooperative children. It was shown that 70%–76% of the children under the age of 5 years accepted this route of administration without any resistance, with only 2.5%–10% resisting this route (Jensen, Schroder & Mansson, 1999; Coventry, Martin & Burke, 1991). Midazolam administered IR has several advantages: it does not depend on child’s cooperation and therefore there is no risk of expectoration (Flaitz & Nowak, 1985). It is relatively less scary and painlessly administered. It has a rapid onset (5–10 min) compared to oral administration (30–90 min) (Lindh-Stromberg, 2001; Fuks et al., 1994; Flaitz & Nowak, 1985), with similar effectiveness to intra-muscular route. The duration of rectal sedation is 60 min compared to 120 min orally (Lindh-Stromberg, 2001; Flaitz & Nowak, 1985), allowing effective sedation with a rapid recovery. Rectal absorption is not subject to delays caused by the digestive tract (presence of stomach content and delayed gastric emptying due to vaso-constrictive effect triggered by fear). Nevertheless, it has been argued that rectal absorption is poor and irregular, since excrement may block penetration of medication to the intestine, causing rejection of the solution and, therefore, reducing reliability (Flaitz & Nowak, 1985; Spear et al., 1991).

Local anesthesia delivery is the most frightening and painful procedure during dental treatment and therefore associated with the highest degree of disruptive behavior when compared to other dental procedures (Gibson et al., 2000; Allen et al., 2002; Palm, Kirkegaard & Poulsen, 2004; Rosenberg, 2002). The computerized controlled local anesthetic delivery system (C-CLAD) (Wand; Milestone Scientific, Inc., Deerfield, IL, USA) was shown to significantly decrease the pain disruptive behavior during buccal, palatal and mandibular block injections (Palm, Kirkegaard & Poulsen, 2004; Rosenberg, 2002; Hochman et al., 1997; Allen et al., 2002; Primosch & Brooks, 2002; Gibson et al., 2000; Saloum et al., 2000; Kudo, 2005; Yesilyurt, Bulut & Taşdemir, 2008; San Martin-Lopez et al., 2005; Grace et al., 2000).

Moreover, 86% of the anxious children prefer the physical appearance of the wand over Citoject (8%) and classic syringe (8%) (Kuşcu & Akyuz, 2006). Since young age and high level of anxiety are both correlated with low level of pain threshold (Lu et al., 2007; Tucker et al., 1989), the objective of the present study was to evaluate the effectiveness of IR midazolam-N_2_O sedation for completion of dental treatment in anxious young uncooperative children when local anesthesia is delivered by a C-CLAD.

## Material and Methods

### Study population

All children (219) who received routine dental treatment under IR midazolam sedation in one pediatric dental clinic (MA) during a three year period and their parents agreed verbally to be enrolled with the present observational study, were included in the study. The studied children were classified as definite negative according to Frankl behavior rating scale and were not able to cooperate under only N_2_O or very young children in pre-cooperative stage (up to 3 years old) (Frankl, Shiere & Fogels, 1962). All children were sedated by IR midazolam (0.4 mg/kg, up to a maximal dose of 7.5 mg) (Malamed, 2004) in conjunctions with N_2_O/oxygen (45%/55%).

Of the 219 treated children (107 boys, 112 girls, mean age 4.3 ± 1.69 y), 53 children (age: 4.12 ± 1.9 y) received three consecutive treatments under sedation in 7- to 10-day intervals. A total of 325 dental treatments under sedation were evaluated.

A structured form was designed to collect demographic and dental variables of each child, including age, gender, behavior during administering of sedation, onset of sedation (time elapse from administering the sedation until agreement to sit in the dental chair), mode of administered local anesthesia (where needed), pain disruptive behavior during administered local anesthesia (McGrath et al., 1985), dental treatment procedure (sealant, restoration, stainless steel crown, pulpotomy, or extraction), behavior during operative treatment (Badalaty et al., 1990), completion of planned treatment, use of restraint during treatment (mouth prop), pulse and saturation before sedation and 5 min after delivery of 100% oxygen with termination of the operative treatment, number of operative treatments performed during each appointment, duration of operative treatment, and any side effects that appeared during treatment. All local anesthetics were administered by C-CLAD (Wand; Milestone Scientific, Inc., Deerfield, IL, USA).

Midazolam (Hoffmann-La Roche Ltd., Basel, Switzerland) was administered in the operating room, after fasting (from midnight, clear drinks were allowed until 2 h before sedation), using a rectal tube (Hoffmann-La Roche Ltd., Basel, Switzerland) dipped in vaseline. All parents gave their verbal consent for administrating the sedation intra-rectally.

In the operating room placid music was played. Once sedation signs appeared (e.g., smiling, relaxation of neck and/or arm), the child was placed in the dental chair. When there was resistance, the child was allowed to wait several more minutes and/or to sit with the parent on the dental chair during N_2_O induction. When the child refused to be alone even after N_2_O induction, his parent was allowed to remain with the child in the dental chair during the entire operative treatment. The Ethics Committee of Tel Aviv University approved the study.

### Sedation monitoring

Each child was continuously monitored for pulse and hemoglobin saturation before midazolam was administered, during treatment and immediately after delivery of 100% oxygen for 5 min with completion of the operative treatment. Continuous monitoring was feasible by stabilizing the monitor-probe on the child-thumb by a sock. Pulse and saturation rate values were recorded every 5 min.

### Computerized administered local anesthesia

Each injection was preceded by an application of topical gel (Benzocaine 20%, Sultan Topex, Englewood, NJ) for 50–60 s. The C-CLAD, with a 30-gauge, extra-short needle was used to administer infiltration or intraligamental injection (Ashkenazi, Blumer & Eli, 2005; Ashkenazi, Blumer & Eli, 2006) and a long needle, 30-gauge, was used to administer mandibular block injection. During intra-sulcular, lingual and long buccal anesthesia, the local anesthesia solution was delivered at the lowest possible velocity throughout the entire injection. During infiltration and mandibular block injection, the local anesthesia solution was delivered at the lowest possible velocity during the first two minutes of injection; afterward the velocity was shifted to regular velocity. Lidocaine cartridges, 2% with 1:100,000 epinephrine (Octacain; Novocal Pharmaceutical of Canada, Cambridge, Ontario, Canada), was used in all procedures. The amount of injected local anesthesia did not exceed 4.4 mg/kg body weight of the child. A rubber dam was applied in most operative treatments.

### Impartial evaluation of pain reaction during administered anesthesia

An impartial observer, not involved in the treatment, studied the children while anesthesia was administered. Behavior during local anesthesia delivery was scored according to the Children’s Hospital of Eastern Ontario Pain Scale (CHEOPS), which refers to the parameters of crying, facial display, verbal expression, torso, and arm and leg movements, and rates them according to possible behaviors: from 0 (behavior that is the antithesis of pain) to 3 (behavior indicative of severe pain) (McGrath et al., 1985). Total scores ranged from 4 to 13. A pilot study was conducted to validate the CHEOPS scale, in which the pediatric dentist and the impartial observer studied 15 patients and rated them separately. Disagreements were discussed until full agreement was achieved. These patients were excluded from the present study.

### Cooperation during sedation

Cooperation during the administration of intra-rectal midazolam was rated as followed: good cooperation (with no crying or movements), crying without resistance, controllable resistance (with or without crying) and violent resistance (with or without crying).

### Sedation evaluation

After completion of the dental treatment, the dentist rated the effectiveness of the sedation using the Houpt-scale (Badalaty et al., 1990).

### Statistical analyses

A chi-square test was used to evaluate categorical variables and one-way ANOVA for continuous variables. Possible associations between behavior during administration of local anesthesia and that during treatment were evaluated using the Spearman correlation test. Repeated measures were used to evaluate the effect of several consecutive treatments on the child’s behavior. A level of *p* < .05 was a priori set as statistically significant.

## Results

### Time to sedation onset

Average onset action time (between intra-rectally administered midazolam until apparent signs of sedation) ranged from 2 to 15 min (155 children, 5.64 ± 2.41 min). Children agreed to sit alone in the dental chair after administering the sedative agent at 3 min (*n* = 24, 15.5%), 4 to 6 min (*n* = 91, 58.7%), 7 to 10 min (*n* = 36, 23.2%) and over 10 min (*n* = 4, 2.6%). After 6 and 10 min, 74% and 97% of the children, respectively, agreed to sit alone or accompanied by their parent.

### Cooperation during sedation

During intra-rectal administered midazolam, good cooperation was shown in 132 children (60.3%), crying without resistance in 21 (9.6%), controllable resistance in 41 (18.7%) and violent resistance in 25 (11.4%).

### Disruptive behavior during injection of local anesthesia (Table S1)

The CHEOPS score during administration of local anesthesia was available for 150 (68.5%) children. Most showed a high level of cooperation (mean 6.69 ± 2.1) on a scale of 4–13, where 4 is the best value: 55% exhibited behavior ranging from 4 to 6, 38% ranged from 7 to 10, and only 7% ranged from 11 to 13 (low level of cooperation). Behavior during administering of local anesthesia was not correlated with cooperation during administering intra-rectal sedation.

### Disruptive behavior during operative treatment according to Houpt scale (Badalaty et al., 1990) (Table S2)

Behavior score during operative treatment was available for 199 children: Most children demonstrated good-to-excellent behavior (4–6, from a range of 1 to 6). The planned treatment was completed in 184 (92.5%), partially completed in 6 (3%), and referred for general anesthesia in 9 (4.5%).

### Dental treatment provided during sedation

Detailed treatments were available for 201 children. Table S3 summarizes the distribution of operative treatments provided in each session of treatment. In most sessions, two operative treatments were performed (excluding sealant). During sedation, 7 children received only sealants. Local anesthesia was administered to 187 children to complete their treatment.

Duration of the operative treatment was available for 153 patients, ranging from 4 to 57 min (mean 27.27 ± 9.46 min). Treatments lasted ≤10 min (3.3%), 11–20 min (20.9%), 21–30 min (45.1%), 31–40 min (23.5%) and >40 min (7.2%).

### Restrain exertion during treatment

During N_2_O inhalation, 164 (74.9%) children sat alone without any passive or active restraints and 55 (25.1%) agreed to sit in the dental chair only when accompanied by their parent (N_2_O inhalation received while seated on their parents’ lap). After induction, 33% of the children, who sat when accompanied by their parent, became significantly more sedated, and therefore, their parent left the dental chair, leaving the child to sit alone without further restraints. Thus, during operative treatment, 182 (83.1%) children sat alone and 37 (16.9%) sat on their parents’ lap. No child was treated with additional passive or active restraints (e.g., papoose board or pedi-wrap) except a mouth prop when needed.

### Pulse and saturation level before and after treatment

At baseline (before administering midazolam), the mean pulse was 92 ± 15.61 and 97.11 ± 14.87 immediately after delivery of 100% oxygen for 5 min; with termination of the operative treatment, 4.44±13.42 beats higher than the pulse at baseline (*p* < .01). No statistically significant changes were noticed in oxygen saturation levels before (98.39 ± 1.22) and immediately after (98.69 ± 1.57) 100% oxygen delivery, with termination of the operative treatment.

### Adverse reactions

Immediately after N_2_O was administered, 3 (1.3%) children had nistagmus, which lasted several minutes. At the end of treatment, immediately after 100% oxygen was administered, 5 (2.3%) children experienced diplopia and 18 (8.2%) had hiccups, both lasting between 5–10 min. One child was scared from the diplopia and become restless with hysterical crying.

### Correlation between child’s behavior during local anesthesia delivery and operative treatment

During local anesthesia, behavior was positively correlated with behavior during treatment. This correlation was applied to each parameter of the behavioral scale, such as sleep (*p* < 0.05), crying (*p* < 0.01), and body movements (*p* < 0.01).

### Effect of age on behavior during local anesthesia and during operative treatment

The child’s overall behavior during operative treatment and movements during treatment showed a weak linear link to their age (${r}_{s}^{1}=.20$, *n* = 192, *p* < 0.05 and ${r}_{s}^{1}=.16$, *n* = 192, *p* < 0.05, respectively). As age increased, the overall behavior during treatment improved and movements during treatment decreased. However, age did not correlate with crying, level of alertness during treatment, or with behavior during local anesthesia.

### Effect of gender on behavior during local anesthesia and during operative treatment

No significant differences were found between genders as to their behavior during local anesthesia or operative treatment, which also applied to the parameters of crying, alertness, and level of movement during treatment.

### Effect of type of operative treatment on behavior during treatment

The child’s overall behavior was significantly better when treatment consisted of sealants and restoration rather than crowns, extractions, and/or pulp therapy (*p* < 0.001).

### Correlation of behavior during treatment and use of a mouth prop

The child’s overall behavior score was significantly worse (*p* < 0.01) when a mouth probe was used during treatment, and there were significantly more movements compared to treatment without a mouth probe (*p* < 0.01).

### Effect of consecutive sedations on behavior during sedation and local anesthesia and during operative treatment

Consecutive sedations had no effect on behavior during administering of the intra-rectal sedation. However, consecutive sedations decreased the degree of the child’s cooperation during treatment and during local anesthesia delivery, although this decrease did not prevent completion of the originally planned treatment. At the third appointment, the behavior score during local anesthesia decreased by 15.7% compared to the second appointment (*p* < 0.05). Similarly, behavior during the third consecutive treatment decreased by 7.4% compared to the first treatment (*p* < 0.05); the body movements score increased by 13.7% (*p* < 0.05), and crying score increased by 9.2% (*p* < 0.01).

## Discussion

The present study showed that midazolam administered intra-rectally in a concentration of 0.4 mg/kg, in combination with C-CLAD, enabled completion of the planned dental treatment in 92% of the uncooperative or very young children. Most children (87%) demonstrated good-to-excellent behavior and only 4.5% were referred for general anesthesia. These results demonstrate improvement as compared to previous published studies which reported 55%–74% good-to-excellent effectiveness in children who received oral or rectal midazolam in concentration of 0.2–1.0 mg/kg but without C-CLAD (Lindh-Stromberg, 2001; Erlandsson et al., 2001; Shapira et al., 1996; Lourenço-Matharu & Roberts, 2010; Nathan & Vargas, 2002).

Improved cooperation of these children was also expressed by the fact that 83% sat alone during treatment with no active or passive physical restraints, and the remainder sat with their parents without any other external restraints (e.g., papoose board or pedi-wrap). This is especially important, since the number of parents who are against the use of physical restraints on their children has increased. Moreover, use of restraints in dentistry, including restraining devices, such as the papoose board, is forbidden by law in some countries.

In addition, in the present study, most children showed a high level of cooperation during local anesthesia delivery (mean score 6.7 ± 2.1, CHEOPS scale). The discrepancy between previous reports regarding disruptive behavior during local anesthesia delivery to the present study may be attributed to the use of C-CLAD in the present study (Allen et al., 2002; Palm, Kirkegaard & Poulsen, 2004; Rosenberg, 2002; Hochman et al., 1997; Allen et al., 2002; Primosch & Brooks, 2002; Gibson et al., 2000; Saloum et al., 2000; Kudo, 2005; Yesilyurt, Bulut & Taşdemir, 2008; San Martin-Lopez et al., 2005; Grace et al., 2000). Interstingly, according to the present results, operative procedures, especially preformed crowns and pulpotomies, caused the highest level of stimuli that evoke disruptive behavior, and not the delivery of local anesthesia. These results are in accordance with Ashkenazi et al. (Ashkenazi, Blumer & Eli, 2005; Ashkenazi, Blumer & Eli, 2006) who have shown that effectiveness of anesthesia was lower when pulp therapy and/or preformed crown were performed.

Sedation did not significantly affect the pulse rate or the percentage of hemoglobin saturation. These findings agree with others (Flaitz & Nowak, 1985; Malamed, 2004) who found that midazolam (0.6 mg/kg) administered rectally is an effective and safe sedative without clinically significant changes in blood pressure or pulse rate.

Nistagmus, hiccups and diplopia were also observed. Nistagmus has been reported (Marhofer et al., 1999) when rectal midazolam (0.3 mg/kg) was used in combination with N_2_O (60%) and in conjunction with haloten (1%–2%). A prevalence of 3.3%–15% was found in children (out of 20 and 30 children, respectively). No report on nistagmus or diplopia could be found after administering midazolam and N_2_O alone. These side effects may reflect opposite unsynchronical relaxation of the extraocular muscles when N_2_O is administered in conjunction with midazolam. To fully understand these effects, further studies are needed to evaluate the presence of these events after midazolam is administered without N_2_O. In the present study, one child cried from the fear of the diplopia. Hiccups have also been reported (Nakai et al., 2000), but the mechanism is unclear.

Many children who are referred for sedation need several consecutive treatments. In the present study, three sequential sedations decreased only slightly the child’s cooperation during administration of local anesthesia and during operative treatments; yet, they did not prevent the completion of the planned operative treatment. It can be concluded therefore, that consecutive sedation treatments are feasible. These results are in accordance with others (Erlandsson et al., 2001; Shapira et al., 1996) who also evaluated the effect of sequential sedations on the child’s level of cooperation during operative treatment. Shapira et al. (1996) found a decreased level of cooperation in the acceptance of the face and nose mask between the first and second treatment, and that none of the children who received midazolam cried or moved (mean score 4) at the first visit as opposed to their reaction at the second appointment (mean 3.5 ± 0.81) (according to Houpt scale). Erlandsson et al. (2001) found that 35% of the children who had more than one treatment showed a change in the level of cooperation in the different sessions, but all treatments were completed.

## Conclusions

IR midazolam sedation in conjunction with N_2_O and C-CLAD is an effective, reliable, and safe method to increase cooperation in very young and/or anxious children who are uncooperative during dental treatment. Hiccups, nistagmus and diplopia, which are transient adverse reactions, may be associated with midazolam. This approach is a preferable alternative to treatment under general anesthesia. Most children change their perception of dental treatment, and even improve their behavior during future dental treatments.

## Supplemental Information

10.7717/peerj.254/supp-1 Table S1 Distribution of children according to their disruptive behavior (Children’s Hospital of Eastern Ontario Pain Scale (CHEOPS) during local anesthesia delivered by a computerized controlled local anesthetic delivery system (C-CLAD) (Wand; Milestone Scientific, Inc., Deerfield, IL, USA)

10.7717/peerj.254/supp-2 Table S2 Distribution of children according to their disruptive behavior (Houpt-scale) during sedation

10.7717/peerj.254/supp-3 Table S3 Distribution of operative treatments provided to 219 sedated children

10.7717/peerj.254/supp-4 Supplemental Information 4 Ethical approval letter
