# Supplementary material for: Success rate of IR midazolam sedation in combination with C-CLAD in pediatric dental patients—a prospective observational study
Source: PeerJ. 2014 Mar 6;2:e254. doi: 10.7717/peerj.254 (PMC3961156; doi:10.7717/peerj.254)
Supplement: Table S1 [file peerj-02-254-s001.docx]

Table 1. Distribution of children according to their disruptive behavior (CHEOPS) during local anesthesia delivered by C-CLAD

| Prevalence (%) | Behavior | Item |
| --- | --- | --- |
| 145 (96) | No crying | Cry |
| 4 (3) | Moaning or crying |  |
| 1 (1) | Screaming |  |
| 90 (62) | Composed | Facial |
| 39 (26) | Grimace |  |
| 18 (12) | Smiling |  |
| 59 (39) | No complaints or other complaints | Child  verbal |
| 28 (19) | Pain complaints with or without other complaints |  |
| 63 (42) | Positive |  |
| 106 (71) | Neutral | Torso |
| 44 (29) | Shifting or tense  or shivering or upright or restrained |  |
| 100 (67) | Not touching | Touch |
| 50 (33) | Reach or touch or grab or restrained |  |
| 123 (82) | Neutral | Legs |
| 27 (18) | Squirming/kicking or drawn up/tensed or standing or restrained |  |
| 150 (100) | Total | |
