# Supplementary material for: Success rate of IR midazolam sedation in combination with C-CLAD in pediatric dental patients—a prospective observational study
Source: PeerJ. 2014 Mar 6;2:e254. doi: 10.7717/peerj.254 (PMC3961156; doi:10.7717/peerj.254)
Supplement: Table S2 [file peerj-02-254-s002.docx]

Table 2. Distribution of children according to their disruptive behavior (Houpt-scale) during sedation

| **Item** | Behavior | Prevalence (%) |
| --- | --- | --- |
| **Sleep** | Fully awake, alert | 155 (78) |
|  | Drowsy, disoriented | 40 (20) |
|  | Asleep | 3 (2) |
| **Crying** | Hysterical crying that interrupts treatment | 12 (6) |
|  | Continuous, persistent crying that makes treatment difficult | 12 (6) |
|  | Intermittent, mild crying that does not interfere with the treatment | 10 (5) |
|  | No crying | 165 (83) |
| **Movements** | Violent movement that interrupts treatment | 36 (18) |
|  | Continuous movement that makes treatment difficult | 38 (19) |
|  | Controlled movement that does not interfere with treatment | 38 (19) |
|  | No movement | 87 (44) |
| **Overall behavior** | **Aborted** - no treatment rendered | 9 (4.5) |
|  | **Poor** - treatment interrupted, only partial treatment completed | 6 (3) |
|  | **Fair –** treatment interrupted but eventually all completed | 10 (5) |
|  | **Good-** difficult, but all treatment performed | 37 (18.5) |
|  | **Very good** – some limited crying or movements | 45 (22.5) |
|  | **Excellent** - No crying or movement | 92 (46) |
| **Total** |  | **199 (100)*** |

*****Data were available for 199 patients
