# Supplementary material for: Success rate of IR midazolam sedation in combination with C-CLAD in pediatric dental patients—a prospective observational study
Source: PeerJ. 2014 Mar 6;2:e254. doi: 10.7717/peerj.254 (PMC3961156; doi:10.7717/peerj.254)
Supplement: Table S3 [file peerj-02-254-s003.docx]

Table 3. Distribution of operative treatments provided to 219 sedated children

| Number of treatments provided per child in each sedation session  ( Range) | Mean number ± SD of treatments provided per child in each sedation-session | Number of sedation sessions in which different types of treatments were provided | Treatment |
| --- | --- | --- | --- |
| 1-8 | 2.44±2.05 | 63 | Sealant |
| 1-5 | 1.76 ± 0.97 | 127 | Restoration (Amalgam or composite) |
| 1-2 | 1.21 ± 0.41 | 67 | Pre-formed crown |
| 1-2 | 1.11±0.32 | 27 | Pulpotomy |
| 1-4 | 1.23±0.65 | 26 | Extraction |
| 1-10 | 2.59±1.80 | 201 | Total operative treatment(including sealant) |
| 1-5 | 1.89±0.98 | 197 | Total operative treatment (excluding sealant) |

* Missing information on 18 sedation-sessions,
