# Supplementary figures and images for: Success rate of IR midazolam sedation in combination with C-CLAD in pediatric dental patients—a prospective observational study
Source: PeerJ. 2014 Mar 6;2:e254. doi: 10.7717/peerj.254 (PMC3961156; doi:10.7717/peerj.254)

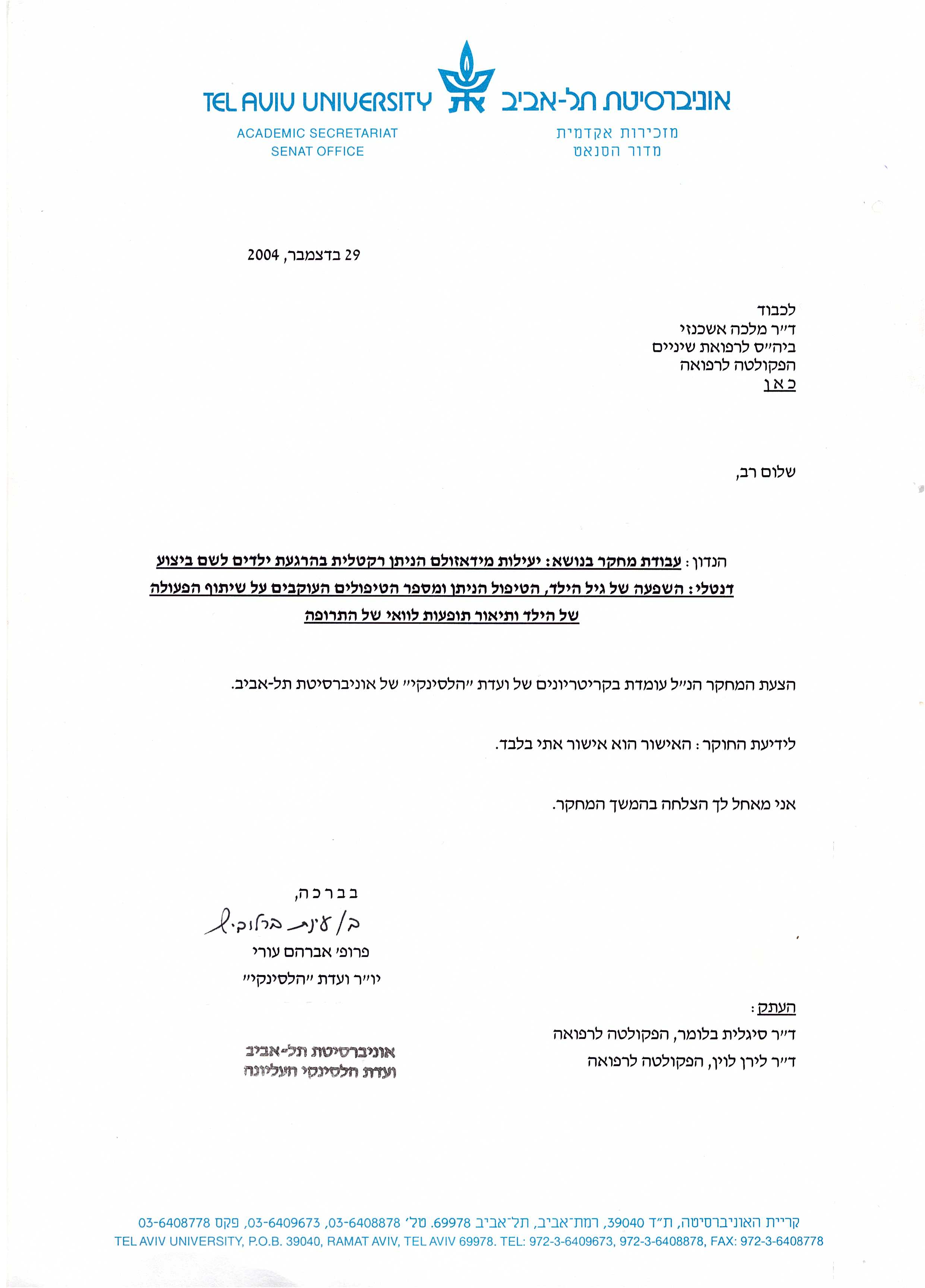

Supplement: Supplemental Information 4 [file peerj-02-254-s004.jpeg]
